# Oral lichen planus and its relationship with systemic diseases. A review of evidence

**DOI:** 10.4317/jced.55145

**Published:** 2018-09-01

**Authors:** Juliana Cassol-Spanemberg, María-Eugenia Rodríguez-de Rivera-Campillo, Eva-María Otero-Rey, Albert Estrugo-Devesa, Enric Jané-Salas, José López-López

**Affiliations:** 1 PhD. Postdoctoral Research Fellow. Specialist in Stomatology and Public Health. Faculty of Medicine and Health Sciences (School of Dentistry), University of Barcelona, Spain; 2 MD, DDS, PhD, Dermatologist and Dentist. Professor of Oral Pathology, Faculty of Medicine and Health Sciences (School of Dentistry), University of Barcelona / Oral Health and Masticatory System Group (Bellvitge Biomedical Research Institute) IDIBELL, University of Barcelona, Spain; 3 DDS, PhD, Odontology. Professor of Master of Daily Practice Dentistry. Department of Stomatology. School of Dentistry. University of Santiago de Compostela, Spain; 4 MD, DDS, PhD. Doctor, Specialist in Stomatology. Professor of Oral Pathology, Faculty of Medicine and Health Sciences (School of Dentistry), University of Barcelona / Oral Health and Masticatory System Group (Bellvitge Biomedical Research Institute) IDIBELL, University of Barcelona, Spain; 5 MD, DDS, PhD. Doctor, Specialist in Stomatology. Professor of Oral Pathology, Faculty of Medicine and Health Sciences (School of Dentistry), University of Barcelona - Head of the Medical Surgical Area and Medical Director of Dentistry Hospital Barcelona University / Oral Health and Masticatory System Group (Bellvitge Biomedical Research Institute) IDIBELL, University of Barcelona, Spain

## Abstract

**Background:**

Oral lichen planus (OLP) is one of the most common dermatological diseases which are present in the oral cavity. It is a chronic autoimmune, mucocutaneous disease that affects the oral mucosa as well as the skin, genital mucosa and other sites.

**Objective:**

Review the relevant information to OLP and its relationship with systemic diseases.

**Material and Methods:**

Searches were carried out in the Medline/PubMed, Lilacs, Bireme, BVS, and SciELO databases by using key-words. After an initial search that provided us with 243 papers, this number was reduced to 78 from the last seven years. One of the first criteria adopted was a selective reading of the abstracts of articles for the elimination of publications that presented less information regarding the subject proposed for this work. All the selected articles were read in their entirety by all of the authors, who came to a consensus about their level of evidence. The Scottish Intercollegiate Guidelines Network (SIGN) criteria were used as the criteria of methodological validation.

**Results:**

Only 9 articles showed an evidence level of 1+, 2+, 3 or 4, as well as a recommendation level of A, B, C or D. Three of them were non-systematic reviews, one was a cohort study and only one was a controlled clinical trial. Three of the studies were case series, with respective sample sizes of 45, 171 and 633 patients.

**Conclusions:**

Several factors have been associated with OLP. Patients with OLP are carriers of a disease with systemic implications and may need the care of a multidisciplinary team. The correct diagnosis of any pathology is critical to making effective treatment and minimizes iatrogenic harm. For OLP is no different, taking into account its association with numerous systemic diseases that require special attention from health professionals. Periodic follow-up of all patients with OLP is recommended.

** Key words:**Oral lichen planus, etiopathogenesis, systemic diseases.

## Introduction

Lichen planus (LP) is a chronic inflammatory mucocutaneous disease that evolves in outbreaks, affecting the skin, mucous membranes or both. It is recurrent and of unknown etiology (1). It tends to adopt different morphologies and experience unpredictable periods of remission and exacerbation (2). It is the dermatological disease that most often presents oral manifestations (3). The exclusive oral presentation of the disease occurs in one out of every three patients, with the three most frequent locations of the buccal mucosa, the tongue and gums (2-4). Oral lichen planus (OLP) may adopt different clinical forms (5,6) and the presentations can be singular or combined. Each one of them has specific features. Its manifestations typically persist for years at a time, alternating between periods of latency and periods of exacerbation (7).

The etiology of this disease remains unknown, but various causal factors have been associated to this disease, among such factors are: anxiety, diabetes, autoimmune diseases, mainly chronic liver disease, intestinal diseases, increased cholesterol, medications, stress, hypertension, infections, contact with dental materials, cancer and a genetic predisposition to cancer (2,3,8-10). Therefore, the diagnosis of OLP must be based on the recognition of the clinical manifestations, as well as the performance of anamnesis in search of a possible cause and effect relationship (7,11). Finally, a histopathological study must be performed in order to enable us to confirm the diagnosis (7).

Based on what has been previously stated, the aim of this paper is to review the information that is relevant to oral lichen planus and its relationship with systemic diseases, as well as briefly review its clinical features and etiology.

## Material and Methods

-Literature Search Strategy

Searches were carried out in the Medline/PubMed, Lilacs, Bireme, BVS, and SciELO databases by using the words: oral lichen planus and systemic diseases, oral lichen planus and hepatitis C virus, diabetes and oral lichen planus, autoimmune diseases and oral lichen planus, chronic diseases and oral lichen planus, intestinal disease and oral lichen planus, cholesterol and oral lichen planus, medications and oral lichen planus, hypertension and oral lichen planus, anxiety and oral lichen planus, stress and oral lichen planus, infections and oral lichen planus in the title and/or abstract. One of the first criteria adopted was a selective reading of the abstracts of articles for the elimination of publications that presented less information regarding the subject proposed for this work.

-Selection, Inclusion Criteria, Data Extraction and Assessment of quality

After an initial search that provided us with 243 papers, this number was reduced to 78 from the last seven years. From there, we used the inclusion criteria of “English and free full text” which gave us a total of 22 papers. These 22 were read in their entirety by all of the authors, who came to a consensus about their level of evidence. Those that were not considered relevant to this review by two or more authors were discarded. The Scottish Intercollegiate Guidelines Network (SIGN) criteria were used as the criteria of methodological validation (12).

## Results

Only 9 of the 22 articles that were reviewed showed an evidence level of 1+, 2+, 3 or 4, as well as a recommendation level of A, B, C or D ( Table 1,  Table 1 continue). Three of them were non-systematic reviews, one was a cohort study and only one was a controlled clinical trial. Three of the studies were case series, with respective sample sizes of 45, 171 and 633 patients.

**Table 1:**
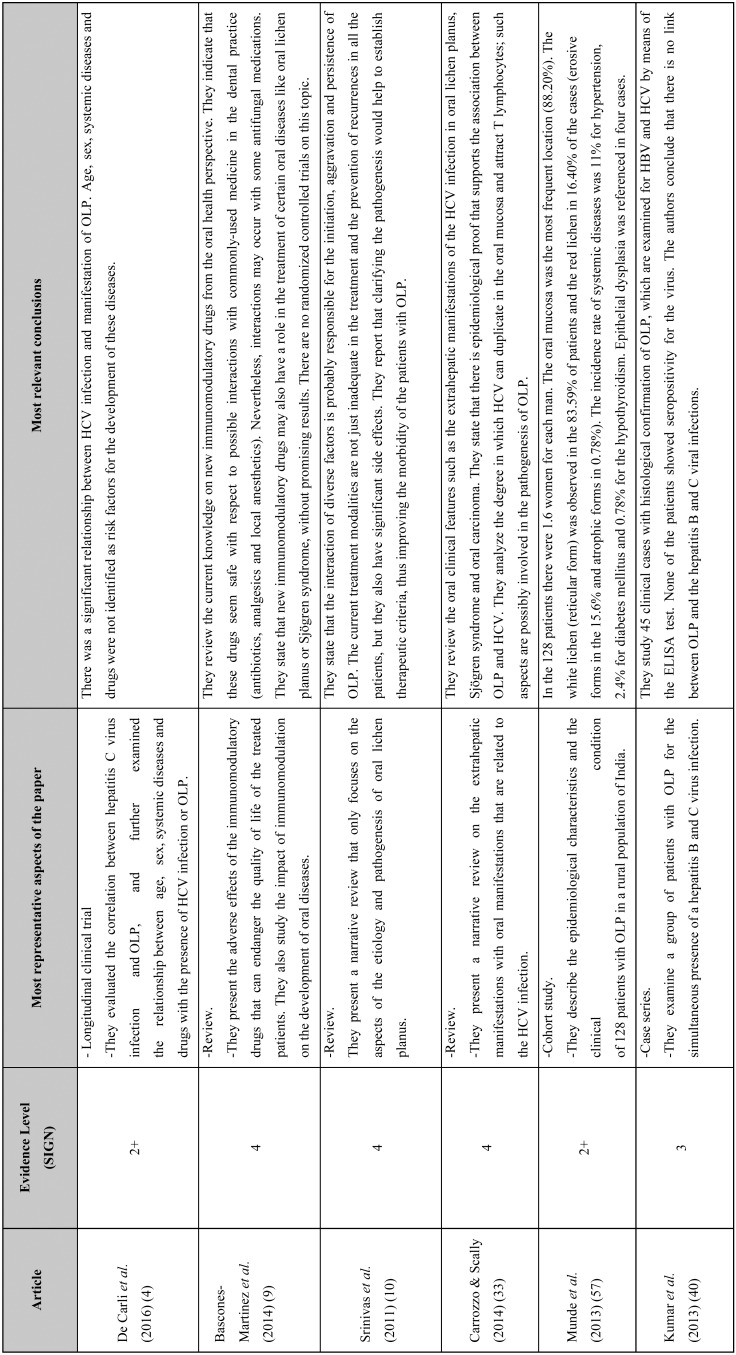
Summary of the eight articles that met the inclusion criteria. The level of evidence for each article is specified. The *Scottish Intercollegiate Guidelines Network* (SIGN) criteria were used as the criteria of methodological validation (12).

**Table 1 continue:**
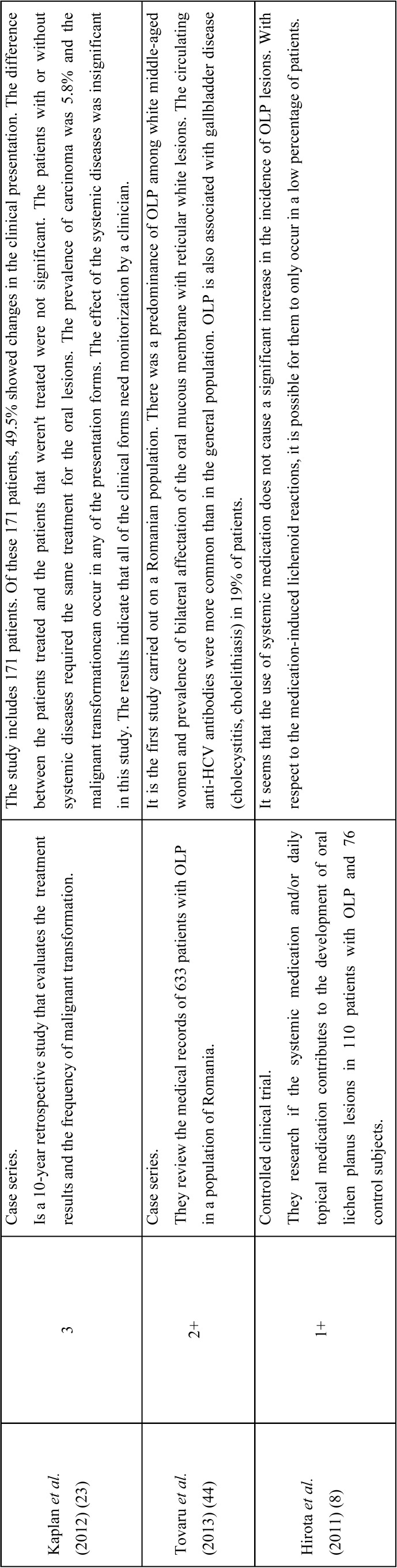
Summary of the eight articles that met the inclusion criteria. The level of evidence for each article is specified. The *Scottish Intercollegiate Guidelines Network* (SIGN) criteria were used as the criteria of methodological validation (12).

## Discussion

For the discussion of the reviewed literature we will use the papers referenced in  Table 1 as well as bibliography of prior interest. We will review the most relevant aspects based on: i) concept and epidemiology, ii) clinical features, iii) etiology and iv) the relationship with systemic diseases.

-Concept and Epidemiology

Lichen planus is a chronic inflammatory dermatosis of autoimmune origin that usually manifests in the oral mucosa. The exact prevalence of OLP is unknown, however different sources report a prevalence of between 0.2% and 5% (13), without racial predominance (14). For every one man, 3 or 4 women are diagnosed with the disease (14), but despite the higher prevalence among women, no link has been found as justification. The hormonal changes caused by the climacteric do not appear to influence its onset, nor the clinical type (15). The typical age range for the manifestation of this disease is between 30 and 70, but cases have been reported in children (16,17).

-Clinical Features

The typical clinical findings related to OLP, especially the presence of a reticular, bilateral and symmetrical pattern, are normally deemed sufficient for the clinical diagnosis of the disease (7). A biopsy allows us to confirm the presumptive clinical diagnosis, also permitting us to rule out areas with signs of cellular atypia and malignancy, which is an aspect that is incompatible with the diagnosis of lichen planus (7).

From a histological perspective, the disease presents a band-like inflammatory infiltrate in the papillary chorion, hyperkeratosis with ortho- and/or parakeratosis and basal layer vacuolation degeneration of the epithelium. All of this leads us to believe that the critical phenomenon in the pathogenesis of this disease is a type of immune aggression directed towards the basal cells of the epithelium (11,18-21).

In regard to its possible malignant transformation, this topic is still controversial (3,11,21). In numerous series such possibility has been proven, although the risk is low (1,6,11,22,23). By modifying some of the above-mentioned criteria, Warnakulasuriya *et al.* (24), in 2007 included OLP as a potentially malignant alteration and the majority of authors recommended the monitoring of these patients for an indefinite period of time, with the aim of early detection of its possible malignization (6,11,21).

Mucosal lesions appear with a fine white reticular pattern (Wickham’s striae), or white papules, in 50-60% of patients. The most frequent manifestation is in the oral and genital mucosa, however lesions can also appear on the skin, scalp, nails, esophagus and eyes (13). It is important to remember that nearly 82% of patients do not show any symptoms, or they report non-specific discomfort such as roughness or a feeling of dryness in the oral mucosa. This implies that the the lesions are many times not perceived by the patient. In most cases the patients are diagnosed for the first time during a routine dental visit. Only 18% of patients experience pain, especially if the lesions are atrophic-erosive, extensive and severe (25).

The lesions are considered atrophic and erosive when they affect the gingiva, and they typically present a clinical pattern known as “chronic desquamative gingivitis” (CDG) (26,27). However, CDG is a non-specific process that may appear in other mucocutaneous diseases, some of which are very relevant, such as cicatricial pemphigoid and pemphigus vulgaris (26). In a study conducted by Bermejo-Fenoll *et al.* (27), in 2010, 314 OLP patients (57.1%) out of a total of 550 had gingival involvement. In their results, 17.5% of women presented desquamative gingivitis, as opposed to 4.7% of men. Mignogna *et al.* (28), in 2000, on the other hand, observed a series of 723 patients with OLP, and identified 336 (48%) cases of gingival involvement, out of which a total of 24 (7.4%) showed severe symptoms.

-Etiology

The etiology of lichen planus remains unknown. The existence of a family history can suggest a possible genetic predisposition (29). Gene polymorphisms of different HLA markers, as well as inflammatory cytokines and chemokines have been associated with the presence of LP. The cause of these polymorphisms, although it is not clear, supports the autoantigen hypothesis (13). Different authors have linked the onset, development and relapse of OLP, to stress, anxiety and depression (30,31). In the pathogenesis there are also phenomenon involved that are of immunological nature, systemic diseases like diabetes, hypertension and chronic liver disease, mainly hepatitis C (14,25,31-33); all of which will be detailed in the following section.

-Relationship with Systemic Diseases

Many studies that have been carried out over the last few years have focused on the relationship between OLP and the hepatitis C virus (HCV) (4,14,25,33,34). Research studies performed in Spain (35), the USA (36), Italy (28), Japan (37), China (38) and Brazil (4,39) have found a significantly greater prevalence of the HCV infection in patients with OLP when compared to the control groups. Lodi *et al.* (2010) (34) published a meta-analysis and in their review they confirmed the association between the HCV infection and lichen planus. Based on this research, patients with LP have a risk that is approximately five times greater than that of the control groups for HCV seropositivity, but if we only focus on patients with OLP, the results are not significant (34). Another interesting fact is that the results that demonstrate this strong association are usually found in geographical areas that are considered hyper endemic areas of HCV. No association has been found in geographical areas with low prevalence of HCV, like India, where a prevalence of 1.8% has been reported (40,41). The general prevalence of liver disease in lichen planus is from 0.1% to 35%, with a greater prevalence in patients that are in their 50s. In these patients the erosive variant of OLP is predominant (52%) and the most reported form of hepatitis is the chronic active presentation (42). The pathogenesis for this association is not clear, but it could be due to the cell-mediated cytotoxicity. Thus, the study performed by Femiano and Scully (2005) (43) suggests the possibility that HCV exerts an indirect effect through the induction of cytokines and lymphokines. Tovaru *et al.* (2013) (44), in a study in which 633 patients with OLP were evaluated, as well as the relationship with liver profiles, found that 24% of the patients with lichen planus showed some type of liver anomaly, and of this group, 9.64% were affected by the hepatitis C virus. In 2006, Lodi (45) carried out a review including data relating to HCV in patients with OLP and he reported high rates of prevalence of HCV in individuals with OLP. There was a significant relationship between HCV infection and manifestation of OLP found by De Carli *et al.* (2016) in a study carried out in Southern Brazil. Age, sex, systemic diseases and drugs were not identified as risk factors for the development of these diseases (4).

The association between lichen planus and cardiovascular risk factors is related to chronic systemic inflammation (46-49). On the other hand, the link between oral lichen planus and dyslipidemia seems to pique the interest of researchers. Various research studies have found a greater prevalence of dyslipidemia in patients with LP, and such studies therefore indicate that the patients with the disease should undergo analytical evaluations (46-49). We must also remember that the presence of dyslipidemia in addition to other risk factors such as hypertension, diabetes mellitus, smoking and kidney disease is very frequent and such factors increase cardiovascular events (46). Lopez-Jornet *et al.*., in 2013 (50) assessed 130 patients with OLP, they found that these patients predominantly suffered from diseases of the musculoskeletal system (22.3%), followed by anxiety and depression (21.5%). Hypertension was observed in 19.2% of the cases, while diabetes type 2 was found in 11.5%. The hypercholesterolemia was found in the 11.5% of patients and hypothyroidism in just 1.5%. The authors sought the association between OLP and autoimmune diseases, but their results did not concur with the previous hypothesis.

The possible link between celiac disease and OLP is backed by Jokinen *et al.* (1998) (51), in their study carried out in 1998. In their research they reveal that of the 39 OLP patients, 22 presented CD positive antibodies. However, Scully *et al.* (1993) (52) did not diagnose celiac disease in any of the 103 patients that were studied, concluding that the association might just be accidental.

As of many years ago it has been suggested that the patients with OLP showed a greater incidence of diabetes than that of the general population (53). The link between OLP and diabetes is controversial, since more than one author (53,54) has indicated that an altered response to the oral administration of glucose exists in patients with LP; since some glycemia curves and insulin responses were obtained that are comparable to the ones that appear in type 2 diabetes. In reference to this matter, Giménez-García and Pérez-Castrillón (2004) (55) found that 10% of the patients assessed in their study were diagnosed with diabetes mellitus and 30% reported a family history of diabetes. We have already mentioned the study by Tovaru *et al.*, in 2013 (44) where they assessed 633 patients with OLP, out of which 10% presented cases of type 2 diabetes. Lundström (1983) (56) found that 28% of the patients with OLP were diabetic, meanwhile in the group of individuals without OLP, only 3% suffered from diabetes. However, Munde *et al.* (2013) (57), found that only 2.4% of their series presented cases of DM. In the population with DM the incidence of lichen planus was 1.6% (58).

The importance that is attributed to psychological factors varies according to the authors. There is controversy about whether or not psychiatric disorders are involved in the genesis of the disease or if it is the result of the presence of chronic painful lesions. In the controlled study carried out by, Hirota *et al.* (2013) (59) the influence of psychological disorders (anxiety and depression) in oral lichen planus was evaluated. The results did not seem to support the idea that anxiety or depression have a role in the development of OLP lesions (59). Rojo-Moreno *et al.* (1998) (30), linked the most symptomatic erosive forms of OLP to stress and anxiety, these authors concluded that patients with oral lichen planus seem to suffer from a greater degree of anxiety and depression. Anxiety or emotional factors would be capable of making the disease chronic, or influencing the apparition of clinical forms that are predominantly red, more symptomatic and more complicated to manage for the clinician (60). Therefore, besides being a possible risk factor, the psychosomatic factors could aggravate the lesions (61). The results of the study carried out by Blanco-Carrión (2002) (31) reflect that psychosomatic alterations, hypercholesterolemia, diabetes and liver disease are frequently associated with patients with OLP. 8.4% of the patients had cutaneous lesions. All of these associations were more frequent in the red clinical forms of oral lichen planus. Anxiety, depression and somatization presented higher values than in the control group; however anxiety had significantly higher values in patients with red lichen. On the other hand, patients with lichen planus reported frequent worsening of their disease throughout periods of stress (7). A study carried out in 1996 by Burkhart *et al.* (1996) (62) proved that 51.4% of the patients with OLP perceived stressful situations in their lives, related to work, personal relationships and losses, which were alterations that occurred before and during the progression of the disease. In this regard, the authors proposed the idea of a link between stress and OLP (62). In 2009, Pokupec *et al.* (63), concluded that specialized attention might be necessary for patients with concomitant psychopathology with respect to lichen planus, particularly those with symptoms of depression, stress and anxiety.

## Conclusions

Oral lichen planus is a chronic mucocuteaneous disease with multifactorial etiology and pathogenesis. Several factors have been associated with OLP. Its association with HCV and other diseases that tend to be linked to LP is controversial and in need of further research.

Clinically speaking, the disease undergoes periods of remission and exacerbation and all patients must be properly monitored. Periodic follow-up of all patients with OLP is recommended. Patients with OLP are carriers of a disease with systemic implications and may need the care of a multidisciplinary team. The correct diagnosis of any pathology is critical to making effective treatment and minimizes iatrogenic harm. For OLP is no different, taking into account its association with numerous systemic diseases that require special attention from health professionals.
